# Geniposide Causes Idiopathic Mesenteric Phlebosclerosis

**DOI:** 10.5152/tjg.2023.23208

**Published:** 2023-08-01

**Authors:** Yoen Young Chuah, Yeong Yeh Lee

**Affiliations:** 1 Division of Gastroenterology and Hepatology, Department of Internal Medicine, Ping Tung Christian Hospital, Taiwan; 2 Department of Nursing, Meiho University, Pingtung, Taiwan; 3 Department of Medicine, University of Sains Malaysia Faculty of Medical Sciences, Kota Bahru, Malaysia

Dear editor,

We read with interest the manuscript by Chou et al^1^ titled “Idiopathic Mesenteric Phlebosclerosis: A Single-Institute Experience in Taiwan,” which was published on February 25, 2023. Idiopathic mesenteric phlebosclerosis (IMP) is a rare form of ischemic bowel, commonly involving the proximal ascending colon, and characterized by wall thickening and serpentine calcifications of mesenteric veins. Asian descents with long-term Chinese herbs ingestion seem to be the most susceptible etiology. Chou et al^1^ reported that 36.1% of 38 patients with IMP had a history of using Chinese medicine. Which Chinese herb is the culprit is often unclear, but extracts of *Gardenia jasminoides* have been reported to be responsible. For example, Hiramatsu et al^2^ showed that 70.4% of IMP patients have exposure to sanshishi, which is an extract of *Gardenia jasminoides*. Yeh et al^3^ reported a case of IMP that was associated with long-term use (8 years) of Chinese medicine, which contained extracts of *Gardenia jasminoides*.

While mechanisms are not entirely clear, it is possible that geniposide, which is the main component of *Gardenia jasminoides*, may be transferred to genipin, which is absorbed into the mesenteric veins and causes intimal hyperplasia, venous wall thickening, and fibrosis, resulting in “mummification.”^4^ Subsequently, the obstruction of venous lumen produces inadequate venous return, intestinal wall thickening and edema, gliosis, and sclerosis and eventually mesenteric phlebosclerosis.^4^ In addition, it is not clear if the disease is impacted by the amount of ingested doses of the *Gardenia jasminoides*. Our case report provides evidence of the daily ingestion of geniposide with the occurrence of IMP.

A 58-year-old female patient was suffering from dull abdominal pain for a week. She went to our emergency department for evaluation. The physical examination was unrevealing and likewise her blood tests. Her pelvic x-ray showed thread-like calcifications (Figure 1A). Bowel thickening and calcifications were also seen in a later computed tomography of the abdomen (Figure 1B). Upon tracing back her drug history, she had been taking a Chinese herbal medicine, Jia Wei Xiao Yao Shan, which contained 95 mg of geniposide (山梔子) (Figure 2, red box) per 1 g in a 3 g powder packet, consistently since January 1, 2000, for 1 pack 1 time per day. Based on the calculation of 1 pack Jia Wei Xiao Yao Shan Chinese medicine per day, a total amount of 2110 g of geniposide had been ingested before the diagnosis of IMP was made on April 13, 2021. 

Our case iterates the potential harms of geniposide in the causation of IMP after a prolonged period of time of ingestion before the occurrence of clinical manifestations and the lack of concerns of patients towards such practices.

## Figures and Tables

**Figure 1.**
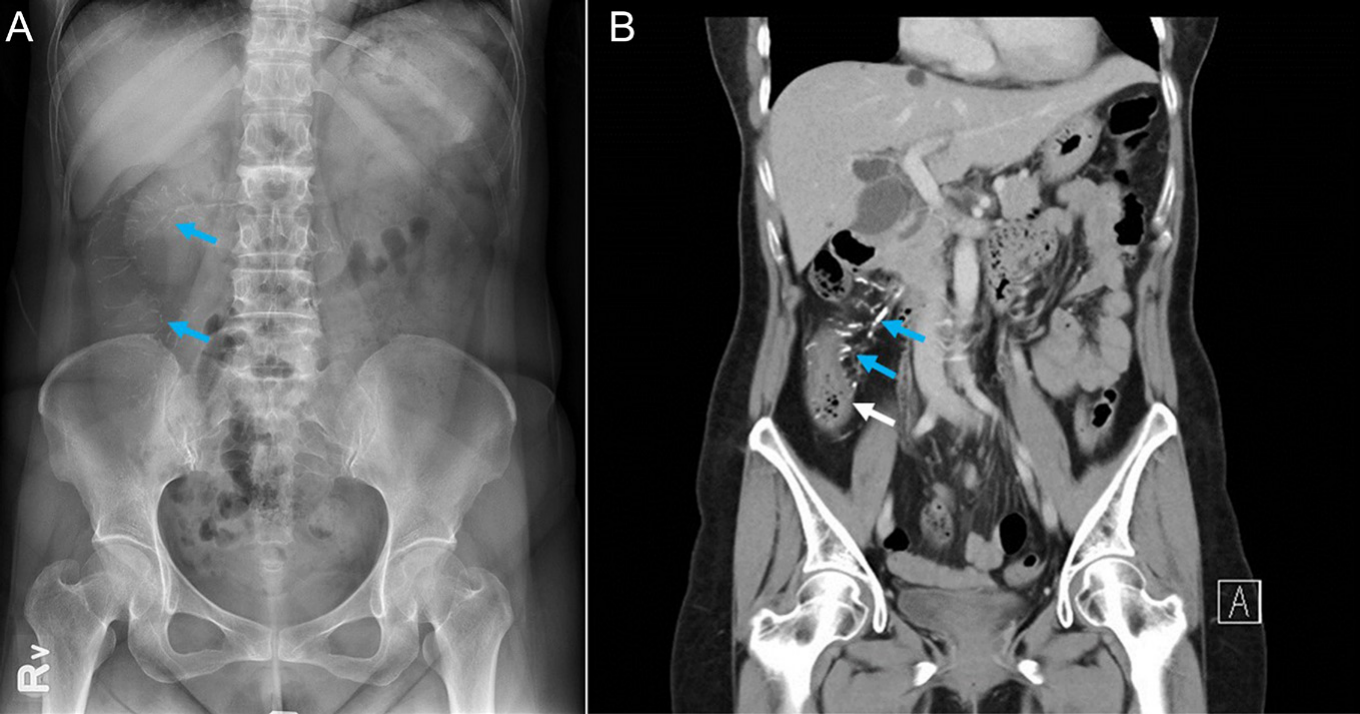
(A) Pelvic X-ray showed thread-like calcifications at ascending colon (blue arrows). (B) Multiple calcifications (blue arrows) are depicted along the thickened wall of ascending colon (white arrow).

**Figure 2.**
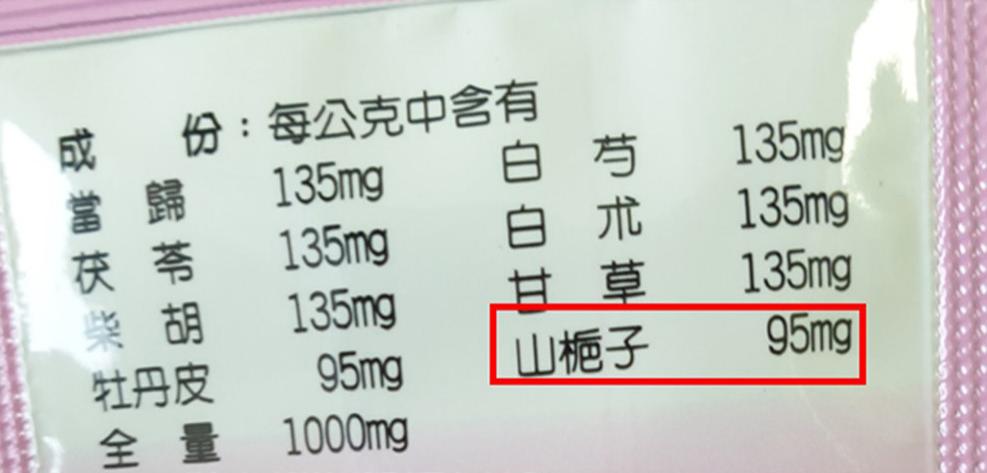
Chinese medicine containing 95 mg geniposide (red box) per 1 g in each packet (3 g in 1 pack).
